# Post-partum paraplegia following spinal anaesthesia: a report of two rare cases

**DOI:** 10.1093/jscr/rjad081

**Published:** 2023-03-09

**Authors:** Tosaddeque Hussain, Indranil Ghosh, Christopher Gerber, Anindya Basu, Mona Tiwari

**Affiliations:** Institute of Neurosciences, Kolkata, India; Advanced Medicare and Research Institute, Kolkata, India; Institute of Neurosciences, Kolkata, India; Institute of Neurosciences, Kolkata, India; Institute of Neurosciences, Kolkata, India

**Keywords:** postpartum, pregnancy, paraplegia, schwannoma, angiolipoma, IDEM, lumbar puncture, spinal anaesthesia

## Abstract

Pregnancy and lumbar puncture are rare instances that can precipitate sudden onset paraplegia in patients with otherwise slow-growing intradural tumours. Surgeons and anaesthesiologists should be aware of the etiological factors leading to pregnancy- and delivery-related rapid tumour growth and its complications. Lumbar puncture-related complications leading to acute precipitation of neurological symptoms must be addressed promptly for favourable outcome in such patients. We describe the report of two patients who developed acute onset paraparesis after spinal anaesthesia for caesarean section. Both were found to be having undiagnosed spinal tumours and managed surgically. We recommend urgent MRI in cases of acute onset non-resolving paraparesis in the peripartum period, for timely diagnosis and management of this rare clinical entity.

## INTRODUCTION

Schwannomas are one of the commonest spinal tumours and account for ~25% cases of intradural tumours [1]. These are benign slow-growing tumours showing gradual onset of symptoms, with a natural history of 6.7 ± 2.7 years [2]. Pregnancy may lead to acute worsening of neurology in previously undiagnosed intradural tumours, because of various factors, as discussed in this report.

## CASE DISCUSSION

### Case 1

**Figure 1:**
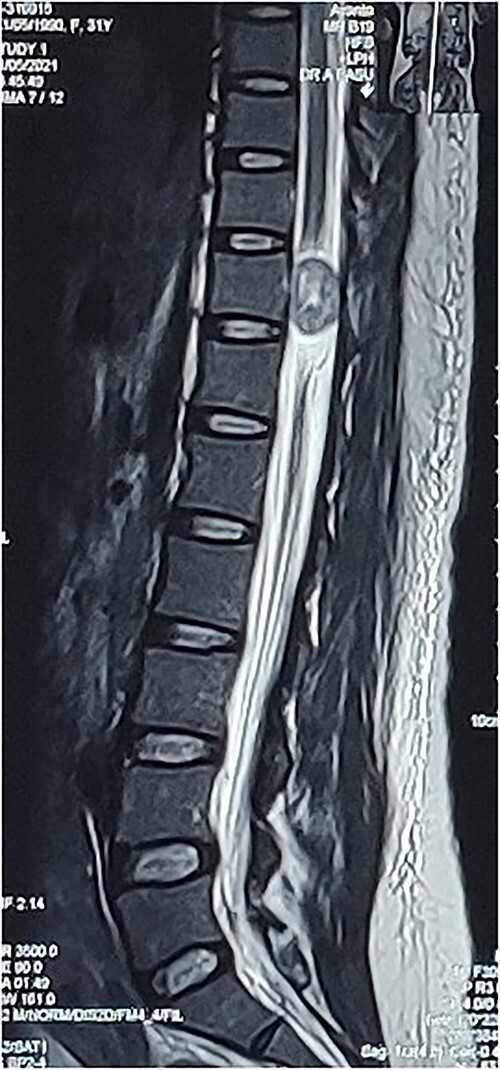
Preop sagittal T2W image showing hyperintense SOL consistent with schwannoma.

A 31-year-old post-partum female presented with acute onset paraparesis on the third day following uneventful caesarean delivery under combined spinal epidural anaesthesia. The patient was referred to our hospital 1 week after delivery with Grade 2 power in left lower limb and Grade 3+ power in right lower limb. There was graded sensory loss below D12 level. Lower limb deep tendon reflexes were exaggerated and bilateral plantar reflexes were extensor. Bladder and bowel were not involved. MAS Grade 1 spasticity was seen in bilateral lower limbs. MRI revealed an ovoid solid lesion behind the D11 vertebral body, which was hypointense in T1W and hyperintense on T2W images (Fig. 1). The following day, she underwent a dorsal laminectomy (D10–11) and the excision of the tumour without any instrumentation. The patient recovered uneventfully from the surgery and had rapid improvement in lower limb muscle strength. On discharge, the patient was independently ambulatory and had Grade 4/5 power in bilateral lower limb. She had improvement in sensation following surgery. On 3-month follow-up, the patient had full motor recovery and was able to walk independently. A repeat MRI showed no residual tumour.

### Case 2

**Figure 2:**
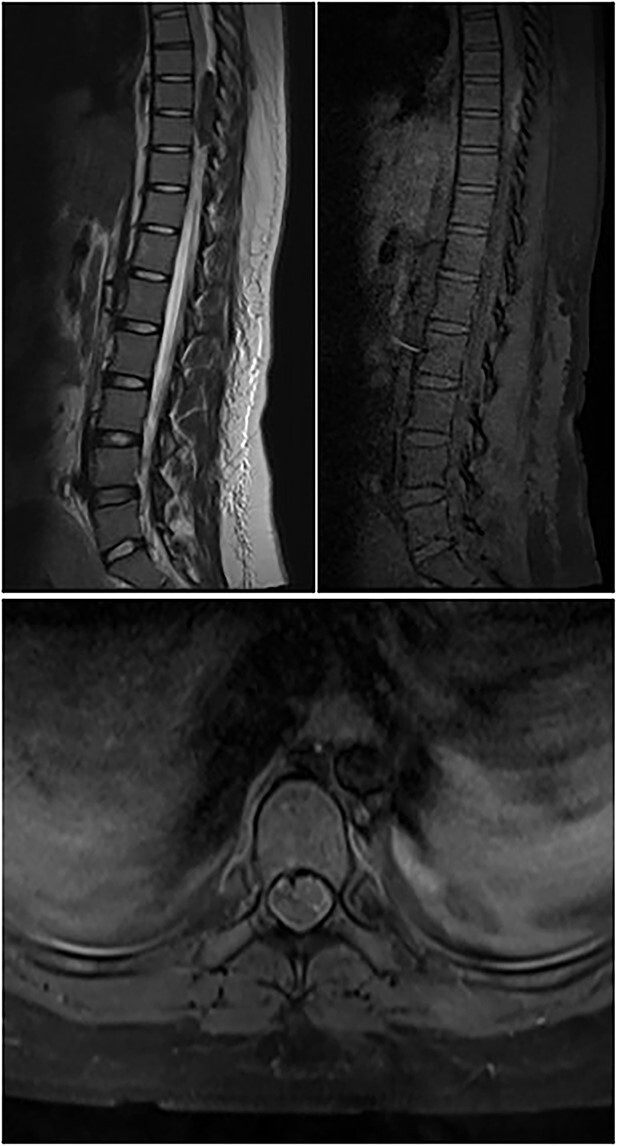
Top left image shows preoperative T2W sagittal MRI of the dorsal spine showing hypointense lesion in the posterior epidural space at D8–9; top right image shows T1W pre-contrast fat-suppressed image showing hyperintensity; bottom image shows post-contrast T1W axial image showing minimal contrast enhancement.

A 23-year-old female presented with acute onset paraparesis following an uneventful caesarean section under spinal anaesthesia. The patient presented to us 3 days after delivery and was paraplegic on examination with a sensory level of D10 and absent deep tendon reflexes in the lower limbs. MRI of the dorsal spine revealed oval well defined T1 hyperintense, T2 hypointense and heterogenous contrast enhancing extradural lesion on the dorsal epidural space at the D8–9 levels compressing the spinal cord and displacing it to the left. Resulting in long-segment cord signal change from D5 to 11 (Fig. 2). The lesion was 4.3 cm in its longitudinal extent and was hypointense on GRE suggestive of blood degradation products. A spinal DSA was performed but did not reveal any abnormality. The patient was operated the following day with D7–9 laminectomy via a posterior approach. Unfortunately, the patient had persistent deficit and continued to remain paraplegic despite extensive rehabilitation. A follow-up MRI scan at 3 months did not reveal any residual tumour.

## DISCUSSION

There are a few instances in the literature reporting cases of acute paraparesis because of IDEM tumours immediately following delivery. It has been hypothesized that spinal tumours grow in size during pregnancy because of pregnancy-related hypervolemia and hormonal changes. Also, the growing foetus causes compression on the IVC causing venous engorgement and neural tissue damage leading to them becoming symptomatic in the peripartum period. Lumbar puncture is another factor that may account for the tumours becoming symptomatic just after delivery under spinal anaesthesia. During spinal anaesthesia, there is a sudden change in CSF pressure because of lumbar puncture, which results in acute cord compression and leads to sudden symptomatic exacerbation in intradural tumours. This may have major medicolegal implications for the anaesthesiologists because of the temporal association of neurological worsening with anaesthesia. Lumbar puncture can cause paraplegia via multiple mechanisms. Vandermeulen *et al*. [3] suggested that, in patients with haemostatic abnormalities, the commonest cause is hematoma. Another mechanism is because of osmotic irritation of the nerve roots because of increased concentration of local anaesthetics [4]. Addition of epinephrine may cause vasoconstriction and ischemic injury to the nerve roots. Kane [5] suggested intraoperative hypotension because of spinal anaesthesia as another cause of ischemic vascular injury to neural structures and thrombosis of spinal arteries. He also suggested multiple lumbar puncture attempts leading to direct nerve injury and hematoma, as possible etiological factors. Fortunately, our patient did not have evidence suggestive of any of the above complications. Mechanism of neurological damage following spinal anaesthesia in underlying spinal space occupying lesions such as tumours have not been well established in literature. Nicholson [6] suggested that the obstruction of CSF flow at the tumour site causes increased local concentration of anaesthetic drugs and consequent toxicity. Hollis [7] suggested that the pressure gradient above and below the tumour following lumbar puncture can result in spinal coning and dangerous neurological complications. It may also exacerbate epidural venous engorgement and impair venous return in the segment below the obstruction caused by the tumour. Indirect evidence from articles discussing the mechanical consequences because of obstruction of CSF flow may further support this theory. Matsubara *et al*. [8] reported a case of bilateral papilloedema associated with a dorsal schwannoma. They suggested that the spinal tumour resulted in the occlusion of CSF flow and increased protein, leading to elevated intracranial pressure and papilloedema. Honda *et al*. [9] reported a case of paraplegia associated with mobile thoracic schwannoma, which was precipitated by myelography. On repeat imaging, the tumour was found to be at a different location from the previous MRI. A second lumbar puncture to relieve CSF pressure led to the improvement of symptoms following which the patient underwent definitive surgery. The above reports hint towards the association between CSF flow and symptomatic spinal tumours, which may partially explain the association with lumbar puncture.

## CONCLUSION

Spinal IDEM tumours may grow rapidly during pregnancy and become symptomatic, requiring urgent surgical decompression to avoid permanent neurological damage. Awareness of their clinical course during pregnancy and post-partum period may help to avoid catastrophic complications such as paraplegia, eclampsia and papilloedema. Knowledge of the possible etiological factors along with timely relevant investigations is the key to successful management of this rare clinical phenomenon.
